# Second-hand smoke exposure in adulthood and lower respiratory health during 20 year follow up in the European Community Respiratory Health Survey

**DOI:** 10.1186/s12931-019-0996-z

**Published:** 2019-02-14

**Authors:** Claudia Flexeder, Jan-Paul Zock, Deborah Jarvis, Giuseppe Verlato, Mario Olivieri, Geza Benke, Michael J. Abramson, Torben Sigsgaard, Cecilie Svanes, Kjell Torén, Dennis Nowak, Rain Jõgi, Jesús Martinez-Moratalla, Pascal Demoly, Christer Janson, Thorarinn Gislason, Roberto Bono, Mathias Holm, Karl A. Franklin, Judith Garcia-Aymerich, Valérie Siroux, Bénédicte Leynaert, Sandra Dorado Arenas, Angelo Guido Corsico, Antonio Pereira-Vega, Nicole Probst-Hensch, Isabel Urrutia Landa, Holger Schulz, Joachim Heinrich

**Affiliations:** 1 Institute of Epidemiology, Helmholtz Zentrum München – German Research Center for Environmental Health, Ingolstädter Landstraße 1, 85764 Neuherberg, Germany; 2 Barcelona Institute for Global Health (ISGlobal), Barcelona, Spain; 3 Universitat Pompeu Fabra (UPF), Barcelona, Spain; 4 CIBER Epidemiología y Salud Pública (CIBERESP), Madrid, Spain; 5 MRC-PHE Centre for Environment and Health, Imperial College London, London, UK; 6 National Heart and Lung Institute, Imperial College London, London, UK; 7 Unit of Epidemiology and Medical Statistics, Department of Diagnostics and Public Health, University of Verona, Verona, Italy; 8 University Hospital of Verona, Verona, Italy; 9 School of Public Health and Preventive Medicine, Monash University, Melbourne, Australia; 10 Department of Public Health, Aarhus University, Aarhus, Denmark; 11 Centre for International Health, University of Bergen, Bergen, Norway; 12 Department of Occupational Medicine, Haukeland University Hospital, Bergen, Norway; 13 Department of Occupational and Environmental Medicine, Sahlgrenska University Hospital, Gothenburg, Sweden; 14 Institute and Outpatient Clinic for Occupational, Social and Environmental Medicine, University Hospital Munich (LMU), Munich, Germany; 15 Comprehensive Pneumology Center Munich (CPC-M), Member of the German Center for Lung Research (DZL), Munich, Germany; 16 Lung Clinic, Tartu University Clinics, Tartu, Estonia; 17 Servicio de Neumología del Complejo, Servicio de Salud de Castilla – La Mancha (SESCAM), Hospitalario Universitario de Albacete, Albacete, Spain; 18 Facultad de Medicina de Albacete, Universidad de Castilla – La Mancha, Albacete, Spain; 19 Department of Pulmonology, Division of Allergy, Hôpital Arnaud de Villeneuve, University Hospital of Montpellier, Montpellier, France; 20 Inserm, Sorbonne Université, Equipe EPAR – IPLESP, Paris, France; 21 Department of Medical Sciences, Respiratory, Allergy and Sleep Research, Uppsala University, Uppsala, Sweden; 22 Department of Sleep, Landspitali National University Hospital of Iceland, Reykjavik, Iceland; 23 Faculty of Medicine, University of Iceland, Reykjavik, Iceland; 24 Department of Public Health and Pediatrics, University of Turin, Turin, Italy; 25 Department of Occupational and Environmental Medicine, Sahlgrenska University Hospital, Gothenburg, Sweden; 26 Department of Surgical and Perioperative Sciences, Surgery, Umea University, Umea, Sweden; 27 Institute for Advanced Biosciences, UGA-Inserm U1209-CNRS UMR 5309, Joint Research Center, Team of Environmental Epidemiology Applied to Reproduction and Respiratory Health, Site Santé – Allée des Alpes, 38700 La Tronche, Grenoble, France; 28 Inserm, UMR 1152, Pathophysiology and Epidemiology of Respiratory Diseases, Paris, France, UMR 1152, University Paris Diderot Paris, Paris, France; 29 Pulmonology Department, Galdakao-Usansolo Hospital, Galdakao, Biscay Spain; 30 Division of Respiratory Diseases, IRCCS Policlinico San Matteo Foundation, Pavia, Italy; 31 Department of Internal Medicine and Therapeutics, University of Pavia, Pavia, Italy; 32 Respiratory and Allergy Clinical Unit, Universitary Hospitalary Complex, Huelva, Spain; 33 Swiss Tropical and Public Health Institute, Basel, Switzerland; 34 Department of Public Health, University of Basel, Basel, Switzerland; 35 Pulmonary Department, Hospital Galdakao, Galdakao, Biscay Spain; 36 Allergy and Lung Health Unit, Melbourne School of Population and Global Health, The University of Melbourne, Melbourne, Australia

**Keywords:** Adults, Smoking, Lung function, Asthma, Respiratory symptoms, Bronchitis, ECRHS

## Abstract

**Background:**

Early life exposure to tobacco smoke has been extensively studied but the role of second-hand smoke (SHS) for new-onset respiratory symptoms and lung function decline in adulthood has not been widely investigated in longitudinal studies. Our aim is to investigate the associations of exposure to SHS in adults with respiratory symptoms, respiratory conditions and lung function over 20 years.

**Methods:**

We used information from 3011 adults from 26 centres in 12 countries who participated in the European Community Respiratory Health Surveys I-III and were never or former smokers at all three surveys. Associations of SHS exposure with respiratory health (asthma symptom score, asthma, chronic bronchitis, COPD) were analysed using generalised linear mixed-effects models adjusted for confounding factors (including sex, age, smoking status, socioeconomic status and allergic sensitisation). Linear mixed-effects models with additional adjustment for height were used to assess the relationships between SHS exposure and lung function levels and decline.

**Results:**

Reported exposure to SHS decreased in all 26 study centres over time. The prevalence of SHS exposure was 38.7% at baseline (1990–1994) and 7.1% after the 20-year follow-up (2008–2011). On average 2.4% of the study participants were not exposed at the first, but were exposed at the third examination. An increase in SHS exposure over time was associated with doctor-diagnosed asthma (odds ratio (OR): 2.7; 95% confidence interval (95%-CI): 1.2–5.9), chronic bronchitis (OR: 4.8; 95%-CI: 1.6–15.0), asthma symptom score (count ratio (CR): 1.9; 95%-CI: 1.2–2.9) and dyspnoea (OR: 2.7; 95%-CI: 1.1–6.7) compared to never exposed to SHS. Associations between increase in SHS exposure and incidence of COPD (OR: 2.0; 95%-CI: 0.6–6.0) or lung function (β: − 49 ml; 95%-CI: -132, 35 for FEV_1_ and β: − 62 ml; 95%-CI: -165, 40 for FVC) were not apparent.

**Conclusion:**

Exposure to second-hand smoke may lead to respiratory symptoms, but this is not accompanied by lung function changes.

**Electronic supplementary material:**

The online version of this article (10.1186/s12931-019-0996-z) contains supplementary material, which is available to authorized users.

## Introduction

Exposure to second-hand smoke remains one of the most common indoor pollutants worldwide. In an overview paper from 2011 as many as 40% of children, 35% of women, and 33% of men were regularly exposed to second-hand smoke indoors worldwide [1]. Children exposed to passive smoke have deficits in lung growth [2–5]. However, the effect of environmental tobacco smoke on respiratory disorders and lung function has not been widely investigated and the associations are less clear in adults [6–8].

Emerging evidence indicates that exposure to second-hand smoke is related to the development of chronic obstructive pulmonary disease (COPD). Based on three studies [8–10], a meta-analysis [11] found an increased relative risk (RR = 1.7, 95% CI: 1.4–2.0) of COPD defined by spirometry in people exposed to passive smoking. A link between exposure to second-hand smoke and an accelerated loss of lung function [6, 8] was suggested, but the evidence is not strong. Results from cross-sectional analyses of data from middle aged adults participating in the European Community Respiratory Health Survey (ECRHS) showed adverse effects of passive smoking on respiratory symptoms including increased bronchial responsiveness, but the negative association with lung function was not statistically significant [12]. In addition, a variety of early life factors including maternal smoking during pregnancy showed an association with asthma and poor lung function in adulthood [13, 14]. A recent report on life-long exposure to tobacco smoke and lung function trajectories to middle age reported accelerated lung function decline in the exposed subjects [15]. However, some research [16, 17] indicates that current or former smokers often suffer from respiratory symptoms, although lung function is still within normal range and the criteria for COPD assessed by spirometry are not met. While there is mounting evidence that second-hand smoke exposure causes respiratory symptoms and lung function deficits at younger ages including young adulthood, the impact in older age groups is less clear.

We aimed to analyse the association of exposure to second-hand smoke with respiratory diseases such as asthma, bronchitis and COPD, asthma-related symptoms and spirometric pulmonary function in long-term follow ups of young and middle aged adults within a large European multicentre study (ECRHS).

## Methods

### Study population

The European Community Respiratory Health Survey (ECRHS) is a multicentre population-based cohort study that began in 1990–1994. Fifty-six centres across Europe and other parts of the world from 25 countries took part. Young adults aged between 20 and 44 years were selected at random from available population-based registers to take part in the survey. It was a two-stage study, with around 200,000 participants in the questionnaire stage 1, and 26,000 in the clinical stage 2. In the follow-up survey (ECRHS II) of the clinical stage 2 more than 10,000 adults from 29 centres in 14 countries participated (1998–2001). Detailed descriptions of the methods for ECRHS I and ECRHS II have previously been published [18, 19]. ECRHS III was the third wave of data collection on the cohort, beginning in 2008. Those who took part in the clinical stages of ECRHS I and II were again contacted, with responders invited to a local fieldwork centre, situated in an outpatient clinic or lung function laboratory. Information was gathered from standardised interviews by well-trained fieldworkers.

The current analyses were restricted to 3011 never and former smoking adults from the random sample who participated in all three surveys and had information on second-hand smoke exposure at all three examinations.

### Definition of smoking and second-hand smoke

At each survey participants were asked “Have you ever smoked for as long as a year?”, and if yes, “Do you smoke now as of one month ago?” Current smokers answered both questions in the affirmative and were excluded. Those who answered the lead question in the negative were classified as never smokers, and ex-smokers were those who answered they had smoked but did not in the last month. Smokers and former smokers were asked about duration of smoking and number of cigarettes smoked per day and pack years were calculated. For analytical purposes smoking status was considered as categorical variables never smoker, ex-smoker with less than 15 pack years and ex-smoker with at least 15 pack years.

Exposure to second-hand smoke was assessed by the question “Have you been regularly exposed to tobacco smoke in the last 12 months?”. Study participants answering in the affirmative were classified as being exposed to second-hand smoke.

### Definition of respiratory health parameters

Information on the following respiratory symptoms and diseases were collected: physician-diagnosed asthma, chronic bronchitis and COPD, as well as on respiratory symptoms such as wheeze, dyspnoea, cough and sputum. The asthma related symptoms were combined in an asthma score [20]. The following criteria for outcome assessment were used:

- Physician-diagnosed asthma: “Have you ever had asthma?” and “Was this confirmed by a doctor?” were answered in the affirmative.
- Asthma symptom score: The sum of positive answers to the following five questions, i.e. the asthma score ranges from 0 to 5, according to Sunyer et al. [20]: 1) “Have you been breathless while wheezing in the last 12 months?”; 2) “Have you been woken up with a feeling of chest tightness in the last 12 months?”; 3) “Have you had an attack of shortness of breath whilst at rest in the last 12 months?”; 4) “Have you had an attack of shortness of breath after activity in the last 12 months?” and 5) “Have you been woken by an attack of shortness of breath in the last 12 months?”
- Nocturnal dyspnoea: “Have you been woken by an attack of shortness of breath at any time during the last twelve months?” was answered in the affirmative.
- Cough: positive answer to at least one of “Have you been woken by an attack of coughing at any time in the last twelve months?”, “Do you usually cough first thing in the morning in the winter?” and “Do you usually cough during the day or night in the winter?”
- Sputum: positive answer to at least one of the following questions: “Do you usually bring up phlegm from your chest first thing in the morning in the winter?” and “Do you usually bring up any phlegm from your chest during the day or at night in the winter?”
- Chronic bronchitis: “Do you usually cough during the day or night on most days for as much as three months per year?” and “Do you usually bring up any phlegm from your chest on most days for as much as three months per year?” were answered in the affirmative.

### Lung function testing

Lung function testing was performed by spirometry during the clinical examination according to the ATS/ERS recommendations [21]. Lung function measures were performed in a sitting position while the subjects were wearing nose clips. At least five, but not more than nine, forced expiratory manoeuvres were performed. The maximum forced expiratory volume in 1 s (FEV_1_) and maximum forced vital capacity (FVC) of the technically acceptable manoeuvres were determined. Spirometric lung function measurements pre-bronchodilation were used in the current analyses. Different spirometers were used across the study centres and follow-up time points within study centres.

Standardised z-scores were calculated based on the reference equations for spirometry from the Global Lung function Initiative (GLI - https://www.ers-education.org/guidelines/global-lung-function-initiative.aspx) [22].

The presence of COPD was based on lung function testing. Study participants with a ratio of FEV_1_ and FVC (measured pre-bronchodilation) below the lower limit of normal (LLN) according to the reference equations for spirometry from the Global Lung function Initiative [22] were classified as COPD patients. It was also defined as the ratio of FEV_1_ and FVC (measured pre-bronchodilation) below 0.7.

### Definition of confounders

Potential confounding variables were assessed by questionnaire or measured at the physical examination. These included sex, age, maternal smoking during pregnancy and/or childhood, paternal smoking during childhood and occupational exposure to dust and fumes. Smoking status was defined as never smoker, ex-smoker with less than 15 pack years and ex-smoker with at least 15 pack years. Socioeconomic status was defined based on the age when fulltime education was completed (less than 17 years, 17 to 20 years and more than 20 years).

Allergen specific IgE was measured at baseline against *D. pteronyssinus*, cat, timothy grass and *Cladosporium* using the Pharmacia CAP System and allergic sensitisation was defined as being sensitised to any of these allergens using a cut-off of 0.35 kUA/L.

Height and weight were measured without shoes and in light clothes at the physical examination.

### Statistical analyses

We modelled the longitudinal impact of changes of second-hand smoke exposure on respiratory health outcomes. The effect of change in second-hand smoke exposure over two examinations on lung function parameters as well as respiratory symptoms and diseases at follow-up was analysed separately for ECRHS I-II, ECRHS II-III and ECRHS I-III. Therefore, study participants were categorised into four groups: those not exposed to second-hand smoke at both examinations (reference category); those not exposed to second-hand smoke at the first examination, but at the second examination (SHS increase); those exposed to second-hand smoke at the first examination, but not at the second examination (SHS decrease) and those exposed to second-hand smoke at both examinations (SHS both). Mixed effects logistic regression models and negative binomial mixed effects models with random intercept for study centre were used for respiratory symptoms/diseases and asthma symptom score, respectively. Linear mixed effects models with random intercept for study centre were used to assess the association of change in second-hand smoke exposure and lung function parameters. All models were adjusted for sex, age, weight, maternal and paternal smoking, exposure to dust/fumes, allergic sensitisation, smoking status and socioeconomic status assessed at baseline and additionally for baseline respiratory symptom/disease and lung function, respectively. The models for the association between change in second-hand smoke exposure and lung function were additionally adjusted for height, weight squared and age squared (to model the non-linear relationship of weight and age with lung function). All continuous covariates were standardised (with mean 0 and variance 1).

The associations between exposure to second-hand smoke at baseline and lung function parameters at the three surveys were analysed to evaluate the effect of second-hand smoke exposure on lung function over time. Therefore, linear mixed effects models were fitted with random intercept for study participants nested in the study centre, and an interaction term between second-hand smoke exposure and time of follow-up, i.e. the time between the particular examinations, was included to model the impact of second-hand smoke exposure on lung function decline [23].

Interaction terms with sex, maternal smoking and paternal smoking were tested. Results from stratified analyses are therefore reported. In addition, sensitivity analyses restricted to never smokers at all three surveys (*n* = 1974 lifetime never smokers) were performed. For the association between change in second-hand smoke exposure over time and lung function at follow-up, additional analyses using percent predicted values according to the Global Lung function Initiative [22] were conducted.

The results for the association between second-hand smoke exposure with respiratory symptoms and diseases are presented as odds ratio (OR) with corresponding 95% confidence interval (CI), whereas the results for the association of second-hand smoke exposure with lung function parameters are presented as regression coefficients (β) with corresponding 95% CI. For the asthma symptom score, the results are presented as count ratio (CR) with corresponding 95% CI.

All analyses were performed using the statistical software R, version 3.4.3 [24], and the R packages “lme4” and “lmerTest”.

## Results

### Description of study population and temporal changes of second-hand smoke exposure and lung function

**Fig. 1:**
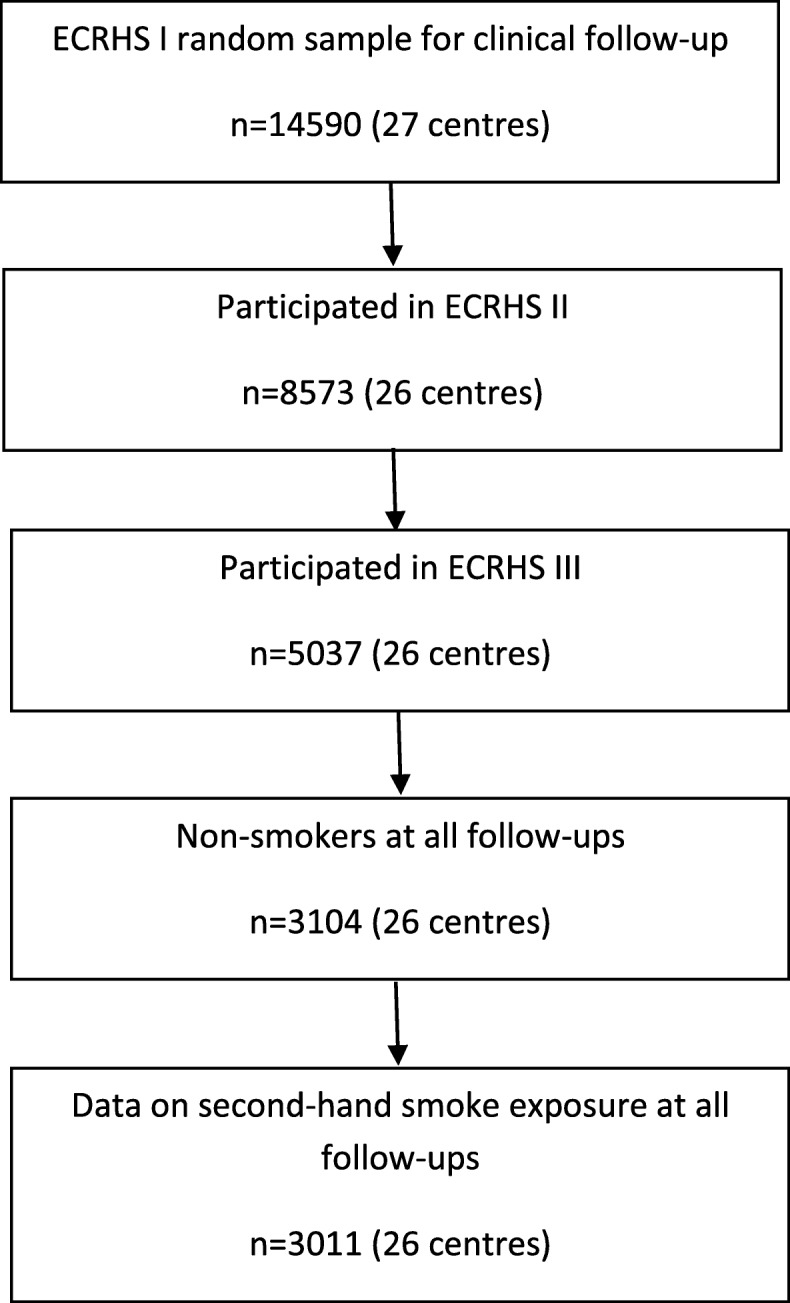
Flow chart of study population

The analyses were based on 3011 non-smoking adults from 26 study centres who participated in all three surveys and had information on second-hand smoke exposure at all three examinations (Fig. 1). The prevalence of reported exposure to second-hand smoke decreased in all participating study centres from ECRHS I to III (Table 1). Overall, at the first examination, 38.7% were exposed to second-hand smoke, 23.0% at the second examination and 7.1% at the third examination. The prevalences were highest in Spain.

**Table 1:** Number of participants and prevalence of second-hand smoke (SHS) exposure by study centre

	n	SHS exposure in ECRHS I, %	SHS exposure in ECRHS II, %	SHS exposure in ECRHS III, %
Antwerp South (Belgium)	104	48.1 (50/104)	32.7 (34/104)	7.7 (8/104)
Antwerp City (Belgium)	99	50.5 (50/99)	36.4 (36/99)	9.1 (9/99)
Hamburg (Germany)	110	46.4 (51/110)	26.4 (29/110)	10.0 (11/110)
Erfurt (Germany)	107	38.3 (41/107)	26.2 (28/107)	6.5 (7/107)
Barcelona (Spain)	54	64.8 (35/54)	40.7 (22/54)	14.8 (8/54)
Galdakao (Spain)	139	74.1 (103/139)	51.1 (71/139)	23.0 (32/139)
Albacete (Spain)	69	68.1 (47/69)	42.0 (29/69)	15.9 (11/69)
Oviedo (Spain)	50	56.0 (28/50)	52.0 (26/50)	28.0 (14/50)
Huelva (Spain)	43	58.1 (25/43)	53.5 (23/43)	18.6 (8/43)
Bordeaux (France)	63	50.8 (32/63)	28.6 (18/63)	6.3 (4/63)
Grenoble (France)	212	34.0 (72/212)	25.5 (54/212)	6.6 (14/212)
Montpellier (France)	88	34.1 (30/88)	13.6 (12/88)	1.1 (1/88)
Paris (France)	186	40.3 (75/186)	33.3 (62/186)	4.8 (9/186)
Pavia (Italy)	51	64.7 (33/51)	47.1 (24/51)	2.0 (1/51)
Turin (Italy)	39	51.3 (20/39)	38.5 (15/39)	12.8 (5/39)
Verona (Italy)	57	35.1 (20/57)	17.5 (10/57)	7.0 (4/57)
Ipswich (UK)	93	31.2 (29/93)	20.4 (19/93)	7.5 (7/93)
Norwich (UK)	88	36.4 (32/88)	11.4 (10/88)	3.4 (3/88)
Reykjavik (Iceland)	197	47.2 (93/197)	26.4 (52/197)	7.1 (14/197)
Bergen (Norway)	186	30.1 (56/186)	11.8 (22/186)	5.4 (10/186)
Gothenburg (Sweden)	149	46.3 (69/149)	6.7 (10/149)	2.7 (4/149)
Umea (Sweden)	162	22.2 (36/162)	8.6 (14/162)	4.9 (8/162)
Uppsala (Sweden)	216	18.1 (39/216)	3.7 (8/216)	1.4 (3/216)
Basel (Switzerland)	221	24.4 (54/221)	16.3 (36/221)	6.8 (15/221)
Melbourne (Australia)	165	13.3 (22/165)	8.5 (14/165)	1.8 (3/165)
Tartu (Estonia)	63	34.9 (22/63)	25.4 (16/63)	3.2 (2/63)
Overall	3011	38.7 (1164/3011)	23.0 (694/3011)	7.1 (215/3011)

The prevalence of respiratory symptoms and diseases and the distribution of lung function parameters and confounding variables in each survey are summarised in Table 2.

**Table 2:** Prevalence of respiratory symptoms and diseases and distribution of lung function parameters and confounding variables^a^

	ECRHS I		ECRHS II		ECRHS III	
	SHS exposed (*n* = 1164)	SHS non-exposed (*n* = 1847)	SHS exposed (*n* = 694)	SHS non-exposed (*n* = 2317)	SHS exposed (*n* = 215)	SHS non-exposed (*n* = 2796)
Sex, female	52.0 (605/1164)	55.5 (1025/1847)	51.9 (360/694)	54.8 (1270/2317)	58.6 (126/215)	53.8 (1504/2796)
Age, years	33.8 (7.5)	35.1 (6.9)	43.5 (7.3)	43.3 (7.1)	53.8 (6.9)	54.7 (7.2)
Age completed full time education						
< 17 years	19.4 (212/1093)	13.8 (232/1686)	24.7 (160/649)	13.3 (284/2130)	29.6 (59/199)	14.9 (385/2580)
17–20 years	39.1 (427/1093)	33.6 (567/1686)	39.8 (258/649)	34.6 (736/2130)	39.7 (79/199)	35.5 (915/2580)
> 20 years	41.5 (454/1093)	52.6 (887/1686)	35.6 (231/649)	52.1 (1110/2130)	30.7 (61/199)	49.6 (1280/2580)
Height, cm	170.4 (9.8)	170.6 (9.5)	169.6 (10.0)	170.9 (9.5)	167.2 (10.2)	170.1 (9.7)
Weight, kg	70.2 (14.0)	68.7 (13.2)	74.5 (15.0)	73.6 (14.9)	77.7 (14.2)	77.6 (16.1)
Paternal smoking	63.3 (714/1128)	58.2 (1059/1819)	67.2 (452/673)	58.1 (1321/2274)	72.9 (153/210)	59.2 (1620/2737)
Maternal smoking	20.5 (237/1155)	20.3 (373/1834)	17.8 (123/690)	21.2 (487/2299)	18.2 (39/214)	20.6 (571/2775)
Allergic sensitisation, IgE	26.5 (267/1007)	33.8 (538/1593)	26.0 (151/581)	32.4 (654/2019)	28.4 (50/176)	31.1 (755/2424)
Dust/fumes exposure	41.8 (484/1159)	35.7 (653/1828)	42.7 (296/694)	39.0 (904/2316)	38.5 (77/200)	22.1 (572/2583)
Ex-smoker	31.2 (363/1164)	28.7 (531/1847)	33.3 (231/694)	29.5 (684/2317)	31.6 (68/215)	29.0 (810/2796)
Smoking status						
Never smoker	69.2 (801/1158)	71.4 (1316/1844)	67.9 (463/682)	73.0 (1633/2236)	70.0 (147/210)	73.3 (1986/2709)
Ex-smoker with < 15 pack-years	23.2 (269/1158)	22.3 (412/1844)	21.6 (147/682)	21.2 (474/2236)	16.2 (34/210)	20.1 (544/2709)
Ex-smoker with > = 15 pack-years	7.6 (88/1158)	6.3 (116/1844)	10.6 (72/682)	5.8 (129/2236)	13.8 (29/210)	6.6 (179/2709)
Asthma, doctor-diagnosed	7.1 (82/1160)	7.4 (136/1846)	10.4 (72/694)	10.8 (250/2312)	15.0 (32/213)	13.8 (385/2792)
Chronic bronchitis	1.4 (16/1162)	1.3 (24/1846)	2.3 (16/693)	1.2 (27/2314)	4.2 (9/215)	2.1 (59/2789)
COPD, FEV_1_/FVC < 0.7	2.7 (29/1078)	3.4 (59/1731)	2.5 (16/630)	4.4 (89/2042)	9.3 (18/193)	10.8 (264/2448)
COPD, FEV_1_/FVC < GLI LLN	4.6 (50/1077)	4.5 (78/1731)	2.2 (14/629)	3.8 (77/2038)	5.7 (11/193)	5.8 (142/2445)
Asthma symptom score						
0	74.8 (866/1157)	76.0 (1392/1831)	67.9 (468/689)	75.0 (1723/2298)	65.6 (137/209)	72.7 (1974/2714)
1	14.1 (163/1157)	13.0 (238/1831)	18.3 (126/689)	13.6 (313/2298)	16.7 (35/209)	16.8 (455/2714)
2	5.7 (66/1157)	5.4 (99/1831)	6.7 (46/689)	5.9 (136/2298)	7.7 (16/209)	6.0 (163/2714)
3	2.9 (33/1157)	2.8 (51/1831)	3.5 (24/689)	3.1 (72/2298)	4.8 (10/209)	2.5 (67/2714)
4	1.6 (18/1157)	1.6 (30/1831)	2.5 (17/689)	1.6 (36/2298)	3.3 (7/209)	1.0 (26/2714)
5	1.0 (11/1157)	1.1 (21/1831)	1.2 (8/689)	0.8 (18/2298)	1.9 (4/209)	1.1 (29/2714)
Dyspnoea	4.6 (54/1163)	4.9 (90/1847)	6.5 (45/694)	5.2 (120/2310)	9.0 (19/211)	5.2 (142/2743)
Cough	33.6 (390/1159)	29.7 (547/1842)	35.3 (244/692)	31.7 (733/2312)	40.1 (85/212)	33.7 (936/2778)
Sputum	11.2 (129/1153)	10.1 (185/1827)	12.0 (83/692)	10.0 (230/2298)	15.2 (32/211)	10.9 (302/2769)
FEV_1_, ml	3800 (824)	3781 (835)	3522 (805)	3595 (819)	3028 (756)	3123 (778)
FVC, ml	4573 (1032)	4591 (1058)	4306 (1000)	4458(1030)	3904 (995)	4063 (993)
FEV_1_/FVC, %	83.5 (6.7)	82.8 (6.5)	82.1 (5.9)	80.9 (6.1)	77.6 (5.6)	77.0 (5.7)

^a^stratified by exposure to second-hand smoke (SHS) and presented as % (n/N) for categorical variables and mean (SD) for continuous variables, respectively

Table 3 shows the change in second-hand smoke exposure from ECRHS I-II, ECRHS II-III as well as ECRHS I-III. Almost 7% (ECRHS I-II) and 2.5% (ECRHS II-III) of the study participants were not exposed at the first, but were exposed at the second examination.

**Table 3:** Second-hand smoke (SHS) exposure in ECRHS I-II, ECRHS II-III and ECRHS I-III^a^

	Changes between ECRHS I and II	Changes between ECRHS II and III	Changes between ECRHS I and III
No SHS exposure at both examinations	54.4 (1639/3011)	74.5 (2243/3011)	58.9 (1774/3011)
No SHS exposure at first examination but at second examination	6.9 (208/3011)	2.5 (74/3011)	2.4 (73/3011)
SHS exposure at first examination but not at second examination	22.5 (678/3011)	18.4 (553/3011)	33.9 (1022/3011)
SHS exposure at both examinations	16.1 (486/3011)	4.7 (141/3011)	4.7 (142/3011)

^a^presented as % (n/N)

The distribution of the lung function parameters as well as the annual decline are summarised in Table 4. All lung function parameters (FEV_1_, FVC and FEV_1_/FVC) decreased over time, with greater decline in the second 10 year follow-up period (ECRHS II-III; 42 ml/year decline in FEV_1_) compared to the first 10 year period (ECRHS I-II; 24 ml/year decline in FEV_1_) as expected with ageing of the population.

**Table 4:** Distribution of lung function parameters and annual change^a^

	ECRHS I	ECRHS II	ECRHS III	Difference between ECRHS I and II (ml or % per year)	Difference between ECRHS II and III (ml or % per year)	Difference between ECRHS I and III (ml or % per year)
FEV_1_ (ml)	3789 (830)	3578 (816)	3116 (777)	−24 (36)	−42 (26)	−34 (17)
FVC (ml)	4584 (1048)	4422 (1025)	4052 (994)	−19 (45)	−34 (35)	−27 (21)
FEV_1_/FVC (%)	83 (7)	81 (6)	77 (6)	−0.2 (0.5)	−0.4 (0.4)	− 0.3 (0.3)

^a^presented as mean (SD)

### Adjusted associations between change in second-hand smoke exposure over time and respiratory symptoms and diseases at follow-up [ECRHS I-II, ECRHS II-III and ECRHS I-III]

**Fig. 2:**
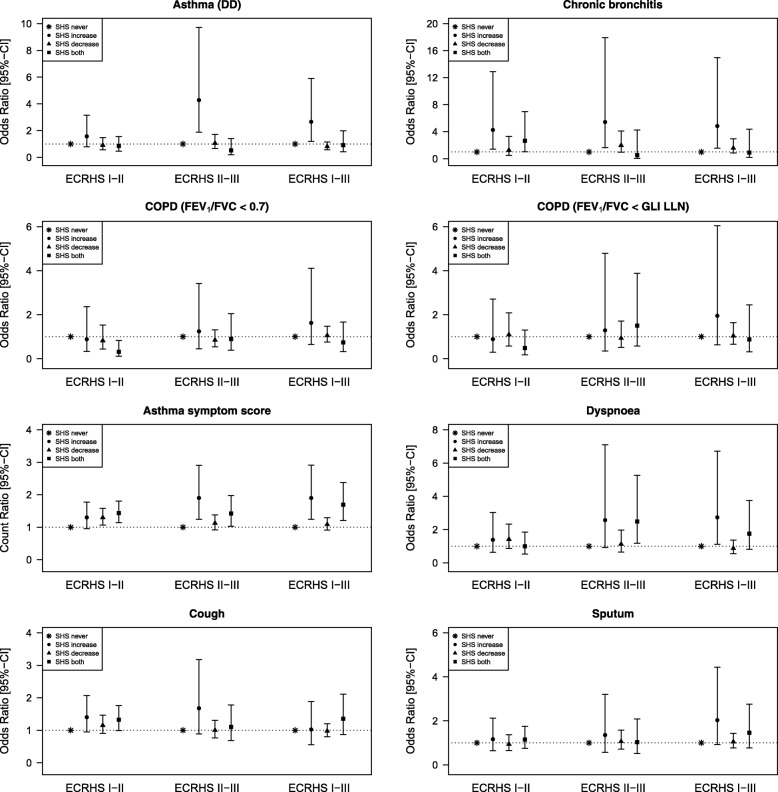
Associations between change in second-hand smoke (SHS) exposure over time and respiratory symptoms/diseases at follow-up. SHS never: no SHS exposure at both examinations (reference category); SHS increase: no SHS exposure at first examination but at second examination; SHS decrease: SHS exposure at first examination but not at second examination; SHS both: SHS exposure at both examinations. All models are adjusted for sex, age, weight, maternal smoking, paternal smoking, combination of smoking status and pack years, education, exposure to dust/fumes, allergic sensitisation (at baseline) and baseline respiratory symptom/disease

Adjusted time variant analysis of second-hand smoke exposure showed that those reporting increased second-hand smoke exposure had increased risks for the development of doctor-diagnosed asthma, chronic bronchitis and increased asthma symptom score, reaching conventional levels of significance from ECRHS II-III as well as from ECRHS I-III overall (Fig. 2). However, compared to those not exposed on both occasions there was no evidence that those reporting exposure on both occasions had an increased risk of asthma or chronic bronchitis. However, asthma score did increase in this group compared to the non-exposed. An increased risk of nocturnal dyspnoea was observed only for those reporting increased second-hand smoke exposure between the first and the third survey but not for those exposed at both surveys.

Spirometrically defined COPD was not statistically significantly associated with changes in second-hand smoke exposure at any of the examined periods over the 20 years after adjustment for several selected confounders (Fig. 2).

### Adjusted associations between change in second-hand smoke exposure over time and lung function parameters at follow-up

There was no association of second-hand smoke exposure with FEV_1_.

**Fig. 3:**
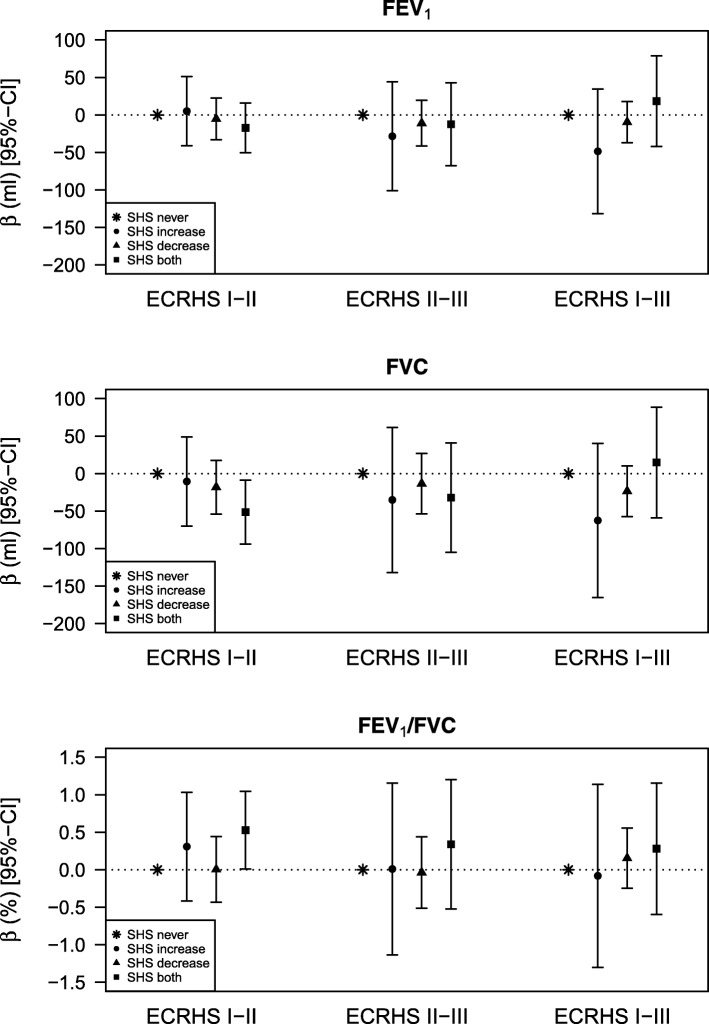
Associations between change in second-hand smoke (SHS) exposure over time and lung function at follow-up. SHS never: no SHS exposure at both examinations (reference category); SHS increase: no SHS exposure at first examination but at second examination; SHS decrease: SHS exposure at first examination but not at second examination; SHS both: SHS exposure at both examinations. All models are adjusted for sex, age, age squared, weight, weight squared, height, maternal smoking, paternal smoking, combination of smoking status and pack years, education, exposure to dust/fumes, allergic sensitisation (at baseline) and baseline lung function

Study participants exposed to second-hand smoke at the first as well as at the second survey (ECRHS I-II) had a reduced forced vital capacity at the second survey (approximately 50 ml) compared to those not exposed to second-hand smoke at these two surveys (Fig. 3) – but there was no clear or consistent pattern of association over the entire study period.

Similarly the ratio of FEV_1_/FVC showed associations with increased second-hand smoke exposure from ECRHS I-II but no consistent pattern when the data were examined over the entire period.

Sensitivity analyses using percent predicted values according to the Global Lung function Initiative showed comparable results (Additional file 1: Table S1).

### Adjusted associations between second-hand smoke exposure at the first examination and lung function as well as lung function decline

The associations of exposure to second-hand smoke at the first examination (ECRHS I) with lung function as well as lung function decline from ECRHS I-III are summarised in Table 5. Exposure to second-hand smoke at the first examination resulted in reduced FEV_1_ and FVC over time, with stronger effects for males compared to females. It also shows that those exposed to second-hand smoke at the first examination had a slightly slower decline in lung function compared to those not exposed.

**Table 5:** Associations between second-hand smoke (SHS) exposure at the first examination and lung function and decline

	Total			Male			Female		
	β	95%-CI	*p*-value	β	95%-CI	*p*-value	β	95%-CI	*p*-value
FEV_1_^a^									
SHS exposure^b^	−44	(−82, −5)	0.03	−75	(− 140, − 11)	0.02	−13	(− 57, 31)	0.56
SHS exposure*follow-up time^c^	1	(0, 3)	0.03	2	(0, 4)	0.03	1	(−1, 3)	0.19
FVC^a^									
SHS exposure^b^	− 56	(−102, − 10)	0.02	− 102	(− 179, − 24)	0.01	−19	(−71, 33)	0.47
SHS exposure*follow-up time^c^	2	(0, 4)	0.02	3	(0, 6)	0.03	2	(0, 4)	0.09
FEV_1_/FVC^a^									
SHS exposure^b^	0.0	(− 0.5, 0.5)	0.96	0.1	(− 0.6, 0.9)	0.71	0.1	(−0.6, 0.7)	0.88
SHS exposure*follow-up time^c^	0.0	(0.0, 0.0)	0.94	0.0	(0.0, 0.0)	0.96	0.0	(0.0, 0.0)	0.62

Models are adjusted for age, age squared, weight, weight squared, height, combination of smoking status and pack years, maternal smoking, paternal smoking, allergic sensitisation, education and exposure to dust/fumes as well as for sex in the total study population

^a^an interaction term between time between follow-ups and SHS exposure is included to determine the effect of SHS exposure on lung function decline

^b^a negative estimate suggests that those exposed to SHS at the first examination had lower average lung function at all three examinations than those not exposed

^c^a negative estimate suggests that those exposed to SHS at the first examination had a higher decline in lung function between the examinations than those not exposed

* indicating the interaction term between SHS exposure and time of follow-up

Sensitivity analyses restricted to lifetime never smokers, i.e. participants who were never smokers at all three surveys showed comparable results (data not shown).

## Discussion

This study investigated the association of exposure to second-hand smoke with respiratory symptoms and diagnoses, as well as lung function and lung function decline in never and former smoking participants in ECRHS I-III. We show that the proportion of the studied population exposed to second-hand smoke fell markedly over the follow-up period of 20 years. Individuals who became exposed to second-hand smoke over time were at increased risk of doctor-diagnosed asthma, chronic bronchitis as well as a higher asthma symptom score. Associations between increase in second-hand smoke exposure and incidence of COPD or lung function were not apparent.

### Comparison with results from other epidemiology studies

Only a few studies have examined the association of exposure to second-hand smoke with onset of asthma in adulthood. In the prospective U.S. Black Women’s Health Study, Coogan et al. observed a positive association of passive smoke exposure with the incidence of adult-onset asthma over 15 years of follow-up in 46,182 women aged 21 to 69 years at baseline [25]. Non-smoking study participants who were exposed to second-hand smoke had a 21% increase (adjusted HR: 1.2; 95%-CI: 1.0–1.5) in asthma incidence compared to those not exposed. Similar findings were observed in two Finnish population-based case-control studies [26, 27]. Exposure to second-hand smoke at the workplace or at home increased the risk for the development of asthma during a period of 2.5 years [27]. In our study, an increase in second-hand smoke exposure over time was associated with an increased risk of doctor-diagnosed asthma. An increased asthma symptom score was observed for those reporting increased second-hand smoke exposure as well as for those exposed on both occasions. A decrease in second-hand smoke exposure was also associated with an increased asthma symptom score. However, this association was not consistent, being only recorded from ECRHS I to ECRHS II, and the strength of the association was rather low. Of note, the ECRHS questionnaire had not been specifically devised to assess changes in respiratory symptoms after smoking cessation or decrease in second-hand smoke exposure. Overall our study findings are consistent with results of the few other studies on second-hand smoke and asthma development in adults.

Previous studies have investigated the association of exposure to second-hand smoke with respiratory symptoms and diseases, especially COPD. For instance, Eisner et al. [6] analysed the effect of lifetime exposure to second-hand smoke on the risk for the development of COPD in 2112 adults (including current, former and never smokers) aged 55 to 75 years in the U.S. It showed a positive significant association between cumulative exposure to second-hand smoke at home (adjusted OR: 1.6; 95%-CI: 1.1–2.2) as well as at work (adjusted OR: 1.4; 95%-CI: 1.0–1.8) with self-reported doctor-diagnosed COPD. A Chinese study [8] also investigated the relationship of self-reported density and duration of exposure to passive smoking with respiratory symptoms (cough, phlegm and shortness of breath) and COPD (FEV_1_/FVC < 0.7 measured pre-bronchodilation) based on data from 15,379 never smoking adults in the Guangzhou Biobank Cohort Study. Exposure to second-hand smoke at home and at work was significantly associated with an increased risk of COPD (adjusted OR: 1.5; 95%-CI: 1.2–1.9) and any respiratory symptoms (adjusted OR: 1.2; 95%-CI: 1.1–1.3).

An increased risk of COPD, defined using the fixed ratio of FEV_1_/FVC < 0.7 measured post-bronchodilation, was seen in those with second-hand smoke exposure in a study [28] of 2182 lifelong never smokers taking part in the Obstructive Lung Disease in Northern Sweden (OLIN) studies. Exposure to second-hand smoke was categorised into several groups based on previous and current exposure to second-hand smoke at home and at work. The strongest associations were seen in those ever exposed at home and at both previous and current work (adjusted OR: 3.8; 95%-CI: 1.3–11.2) as well as for those currently exposed at home and at both previous and current work (adjusted OR: 5.7; 95%-CI: 1.5–22.5). A significant dose dependent relationship of exposure to second-hand smoke with mortality from different diseases, including COPD amongst other causes of death, could be shown in another study [10]. In contrast, a study conducted by Chan-Yeung et al. [9] found no association between exposure to second-hand smoke and an increased risk for COPD in a small sex- and age-matched case-control study comprising 289 patients and controls, respectively, in Hong Kong, China.

The different associations between exposure to second-hand smoke and COPD in the above studies might be due to the different definition of COPD as some studies used questionnaire-based information whereas others used spirometric measurements. Furthermore, some studies were restricted to lifetime never smokers compared to studies also including active smokers. We have shown no significant association between increase in second-hand smoke exposure and incidence of COPD based on lung function testing in our study, which was restricted to never and former smokers. The different observed associations might also be due to residual confounding in some studies or potential misclassification of self-reported exposure to second-hand smoke in our study.

Another study, based on Taiwan’s National Health Insurance Bureau claims data, investigated the association of exposure to second-hand smoke and chronic bronchitis in women [29] and showed that women who were exposed to second-hand smoke had a 3.7 (95%-CI: 1.2–11.3) higher risk of chronic bronchitis compared to those not exposed to second-hand smoke. Furthermore, exposure to second-hand smoke was also associated with mild (adjusted OR: 1.8; 95%-CI: 1.1–2.9) and moderate (adjusted OR: 3.8; 95%-CI: 1.7–8.6) COPD as defined by GOLD.

We have shown a significant positive association of new exposure to second-hand smoke between two surveys with chronic bronchitis at follow-up, defined as having cough and sputum. However there was no evidence of a decrease in chronic bronchitis if exposure to second-hand smoke stopped over the same time frame. In addition, exposure to second-hand smoke was not associated with COPD defined by a ratio of FEV_1_/FVC below 0.7 [30, 31]. According to the original classification of COPD from the Global Initiative for Chronic Obstructive Lung Disease (GOLD) in 2001 [32], stage 0 “at risk” is characterised by chronic symptoms (sputum production and cough) with still normal spirometry, i.e. the ratio between FEV_1_ and FVC of at least 0.7. This GOLD stage 0 would be similar to the definition of chronic bronchitis used in this analysis which requires a positive answer to both the question on cough and the question on sputum, independent of lung function. Moreover, the overlap between chronic bronchitis and COPD defined by spirometry was quite small in this study. As an effect of exposure to second-hand smoke was found in this study only for chronic bronchitis, but not for COPD, one might speculate that these results indicate a transient effect, but not structural changes in the airways as would be common in COPD patients.

Although COPD is generally considered a disease characterised by a progressive, gradually accelerating decline in FEV_1_ Macklem has pointed out that increase in residual volume (RV) is the first functional abnormality in chronic bronchitis [33]. Thus, gas trapping with reduction of FVC is an early abnormality because RV increases more than the total lung capacity (TLC). The observed decrease in FEV_1_ occurs because of a reduction in FVC. The FEV_1_/FVC ratio will decrease because of loss of lung elastic recoil, a *sine qua non* of COPD [33, 34] but early in the disease the decrease in FVC may exceed that in FEV_1_ with a paradoxical effect on the FEV_1_/FVC ratio. This may explain why we did not see an association with COPD defined by spirometric lung function parameters.

A reduced FEV_1_ and FVC over time was observed for those reporting exposure to second-hand smoke at the first examination, with stronger effects for males compared to females. Several studies have investigated the association between second-hand smoke exposure and lung function, suggesting sex differences in vulnerability [35–38]. However, the findings are inconsistent. Some studies found adverse effects of passive smoking on spirometric lung function parameters for both sexes [35, 38], whereas another study found stronger effects for women compared to men [36]. Our results are consistent with findings of the study conducted by Masjedi et al. [37] showing a negative association between second-hand smoke exposure and lung function among men, but not among women.

Janson et al. [39] investigated changes and determinants for changes in active as well as passive smoking in the first and second survey of the European Community Respiratory Health Survey showing that exposure to second-hand smoke was higher among subjects with lower socio-economic status and educational level. Furthermore, subjects exposed to second-hand smoke were less likely to quit smoking suggesting that a decrease in second-hand smoke exposure might be effective in decreasing active smoking. In our study, exposure to second-hand smoke decreased during the 20 years of follow-up where for many of the study centres the decrease between the second 10 year follow-up period was stronger compared to the first 10 year period. However, second-hand smoke exposure was still present in all participating centres.

### Strengths and limitations

The European Community Respiratory Health Survey has a longitudinal study design with two follow-ups approximately 10 and 20 years, respectively, after the first survey and therefore we can model the association between changes in second-hand smoke exposure over time with respiratory health outcomes. We are also able to investigate the effect of exposure to second-hand smoke at baseline on lung function decline using spirometric measurements that were performed and quality controlled according to well established guidelines. Also the large study population of around 2000 never-smoking study participants and the high number of participating centres and countries are further strengths.

However this study has some limitations. The information on second-hand smoke exposure as well as respiratory symptoms and diseases was obtained using self-administered questionnaires completed at follow-up and no biomarkers for exposure to second-hand smoke were available. Moreover, the information on second-hand some exposure was only requested for the last 12 months at each survey and not for the total study period. Furthermore, information on the number of cigarettes smoked by other people was not available. No dose-related association between second-hand smoke exposure and respiratory health has been investigated which has to be taken into account when drawing conclusions.

In addition, against a backdrop of falling smoking rates and smoke free legislation across Europe only a small proportion of the study group became newly exposed to second-hand smoke over the period of the study. Questionnaire-based information on second-hand smoke exposure might be prone to reporting bias as subjects having respiratory symptoms or diseases might tend to be more affected. Siroux et al. [40] has found no indication that asthma status influences reporting of exposure to second-hand smoke in childhood or adulthood but we cannot exclude that our results are related to reporting biases. The use of different spirometers across study centres and surveys could have resulted in temporal differences in lung function measurements. Sensitivity analyses using lung function values corrected for this change showed comparable results. Furthermore, it was difficult to disentangle the survey and age effects due to three time points comprising two follow-ups each after approximately 10 years as different findings were observed for the association of new exposure to second-hand smoke between two surveys with respiratory symptoms and diseases at follow-up.

The questions on cough and sputum were only requested for winter and not for summer, as these symptoms are often more worse during the winter months. Furthermore, no information on the change of the ventilation equipment used for air cleaning during the follow-up periods was available and thus could not be considered as potential confounding variable.

## Conclusion

In a longitudinal analysis of adults, following a multi-centre cohort over twenty years, exposure to second-hand smoke decreased substantially during the study period. Second-hand smoke exposure in adults was associated with an increased risk for asthma and chronic bronchitis. Our results support further restrictions on smoking in public places.

## Additional file

Additional file 1: **Table S1.** Associations between change in second-hand smoke (SHS) exposure over time and lung function at follow-up [percent predicted values according to the Global Lung function Initiative – GLI]. (DOCX 18 kb)
