# Supplementary material for: Second-hand smoke exposure in adulthood and lower respiratory health during 20 year follow up in the European Community Respiratory Health Survey
Source: Respir Res. 2019 Feb 14;20:33. doi: 10.1186/s12931-019-0996-z (PMC6376659; doi:10.1186/s12931-019-0996-z)
Supplement: Supplementary file 1 — Table S1. Associations between change in second-hand smoke (SHS) exposure over time and lung function at follow-up [percent predicted values according to the Global Lung function Initiative – GLI]. (DOCX 18 kb) [file 12931_2019_996_MOESM1_ESM.docx]

# Additional file

Additional file 1: Table S1. Associations between change in second-hand smoke (SHS) exposure over time and lung function at follow-up [percent predicted values according to the Global Lung function Initiative – GLI]

|  |  | **change ECRHS I-II** | | |  | **change ECRHS II-III** | | |  | **change ECRHS I-III** | | |
| --- | --- | --- | --- | --- | --- | --- | --- | --- | --- | --- | --- | --- |
|  |  | β | 95%-CI | p-value |  | β | 95%-CI | p-value |  | β | 95%-CI | p-value |
| **FEV_1_ pp (GLI)** | |  |  |  |  |  |  |  |  |  |  |  |
|  | SHS increase | 0.1 | (-1.2, 1.4) | 0.91 |  | -1.1 | (-3.4, 1.2) | 0.36 |  | -1.9 | (-4.5, 0.6) | 0.14 |
|  | SHS decrease | -0.1 | (-0.9, 0.7) | 0.80 |  | -0.3 | (-1.2, 0.7) | 0.59 |  | 0.1 | (-0.8, 0.9) | 0.90 |
|  | SHS both | -0.3 | (-1.2, 0.7) | 0.58 |  | -0.4 | (-2.1, 1.4) | 0.69 |  | 0.8 | (-1.1, 2.6) | 0.42 |
|  |  |  |  |  |  |  |  |  |  |  |  |  |
| **FVC pp (GLI)** | |  |  |  |  |  |  |  |  |  |  |  |
|  | SHS increase | -0.4 | (-1.7, 0.9) | 0.56 |  | -1.0 | (-3.4, 1.4) | 0.41 |  | -2.3 | (-4.8, 0.2) | 0.08 |
|  | SHS decrease | -0.3 | (-1.1, 0.5) | 0.45 |  | -0.2 | (-1.2, 0.8) | 0.70 |  | -0.3 | (-1.1, 0.6) | 0.53 |
|  | SHS both | -1.0 | (-1.9, -0.0) | 0.04 |  | -0.9 | (-2.7, 0.9) | 0.33 |  | 0.4 | (-1.4, 2.3) | 0.64 |
|  |  |  |  |  |  |  |  |  |  |  |  |  |
| **FEV_1_/FVC pp (GLI)** | |  |  |  |  |  |  |  |  |  |  |  |
|  | SHS increase | 0.4 | (-0.5, 1.3) | 0.36 |  | 0.0 | (-1.5, 1.4) | 0.99 |  | 0.1 | (-1.4, 1.7) | 0.86 |
|  | SHS decrease | 0.0 | (-0.5, 0.5) | 1.00 |  | 0.0 | (-0.6, 0.6) | 0.98 |  | 0.3 | (-0.2, 0.8) | 0.26 |
|  | SHS both | 0.6 | (-0.0, 1.3) | 0.05 |  | 0.5 | (-0.6, 1.6) | 0.40 |  | 0.5 | (-0.7, 1.6) | 0.42 |
|  |  |  |  |  |  |  |  |  |  |  |  |  |
| reference category: no SHS exposure at both examinations | | | | | | | | | | | | |
| SHS increase: no SHS exposure at first examination but at second examination; SHS decrease: SHS exposure at first examination but not at second examination; SHS both: SHS exposure at both examinations | | | | | | | | | | | | |
| adjusted for weight, weight squared, maternal smoking, paternal smoking, combination of smoking status and pack years, education, exposure to dust/fumes, allergic sensitisation (at baseline) and baseline lung function (percent predicted values according to GLI) | | | | | | | | | | | | |
